# The Strongylidae belonging to *Strongylus* genus in horses from southeastern Poland

**DOI:** 10.1007/s00436-012-3087-3

**Published:** 2012-09-08

**Authors:** M. B. Studzińska, K. Tomczuk, M. Demkowska-Kutrzepa, K. Szczepaniak

**Affiliations:** Department of Parasitology and Invasive Diseases, Faculty of Veterinary Medicine, University of Life Sciences in Lublin, Akademicka 12, 20-033 Lublin, Poland

## Abstract

Postmortem parasitic examinations of the large intestines of 725 slaughtered horses from individual farmers in southeastern Poland were carried out. The examinations were carried out monthly since February 2006 until January 2007 (except for August 2007 because of a technological stoppage in the slaughterhouse). The examinations included the intensiveness and extensiveness of the infestation of the Strongylidae belonging to the *Strongylus* genus. The Strongylidae were found in 26.5 % of the examined horses. *Strongylus vulgaris* was the most dominant nematode and had a 22.8 % prevalence, *Strongylus edentatus* was carried by 18.3 % of the horses. *Strongylus equinus* was identified only in 1.7 % of the examined horses. Our findings revealed that combined infestation of *S. vulgaris* and *S. edentatus* occurred in 100 (52.1 %) of the 725 horses infected by the Strongylidae. The present results indicate that the lowest prevalence of strongyle species except for *S. equinus* was found in January, February, and March. However, it is difficult to draw a conclusion because of an extremely low extensiveness of infestation. The results indicate that the prevalence of the Strongylidae in horses from southeastern Poland is limited.

## Introduction

Reports of other authors and our earlier coproscopic and postmortem examinations revealed that the occurrence of nematode parasites from the family Strongylidae is common in horses from both studs and individual farms in southeastern Poland (Gawor 1995, 2000, 2002; Gawor et al. 2006; Gundłach et al. 2004; Kornaś et al. 2004a; Kornaś et al. 2004b; Sobieszewski 1967). Customarily, the Strongylidae fall into the subfamily Strongylinae (15 species organized in five genera: *Strongylus*, *Triodontophorus*, *Bidentostomum*, *Craterostomum*, and *Oesophagodontus*, grouped as large strongyle species) and Cyathostominae (52 species organized in 14 genera including *Cyathostomum*, *Coronocyclus*, *Cylicodontophorus*, *Cylicocyclus*, *Cylicostephanus*, *Poteriostomum*, *Gyalocephalus*, grouped as small strongyle species) (Lichtenfels et al. 2003). They differ morphologically and reveal various developmental cycles, localizations, feeding manner, pathogenicity of mature and larva forms, and resistance to drugs. In spite of numerous reports on horse parasites, detailed data including nematode parasites belonging to the genus *Strongylus* considered as especially pathogenic are scarce.

It should be stressed that in the case of the genus *Strongylus*, both mature and larva forms are pathogenic (Gundłach and Sadzikowski 2004; Schnieder 2006) among which *Strongylus vulgaris* larvae migrating in the circulatory system are regarded as the most pathogenic. These larvae produce adhering to wall clots and aneurysms, and emboli in the small arterioles. The larva *Strongylus edentatus* which migrates under the parietal peritoneum may cause peritoneum inflammation. It is believed that the larvae migrating through the liver may damage this organ. Similarly, *Strongylus equinus* larvae may also damage the liver and then cause inflammation of the pancreas. Mature nematodes may sponge and cause lesions in the large intestine by fixating with large mouth capsules to the mucous membranes. The nematodes fitted with cutting components produce incisions in the mucous membranes and take up the blood, which may lead to anemia in the case of a great number of larvae. The above findings are supported by our observation.

The manner of feeding of strongylids from the genus *Strongylus* seems to differ from that of small strongyle species belonging to the subfamily Cyathostominae (Gawor 2006). The small strongyle species harbor in the contents of large intestine and localize in its parts depending on the species. These nematodes feed mainly on protozoa of the occupied part of the intestine and also small plant elements. Thus, mature small strongyles do not damage significantly the mucosal membrane of the intestine but may impair digestion by changing the population of protozoa.

The aim of the present studies was to provide additional information involving the infestation of strongyles from the genus *Strongylus* in horses.

## Material and methods

The large intestines collected from 725 slaughter horses from individual farms in southeastern Poland were autopsied for parasitic examinations. The examinations were carried out monthly since February 2006 until January 2007 (except for August 2007 because of a technological stoppage in the slaughterhouse). At necropsy, care was taken on localization of strongyles and lesions caused by the nematodes. The nematodes isolated from the intestine were differentiated on the basis of detailed anatomical structure. The number of strongyles belonging to the genus *Strongylus* was evaluated.

## Results

**Fig. 1:**
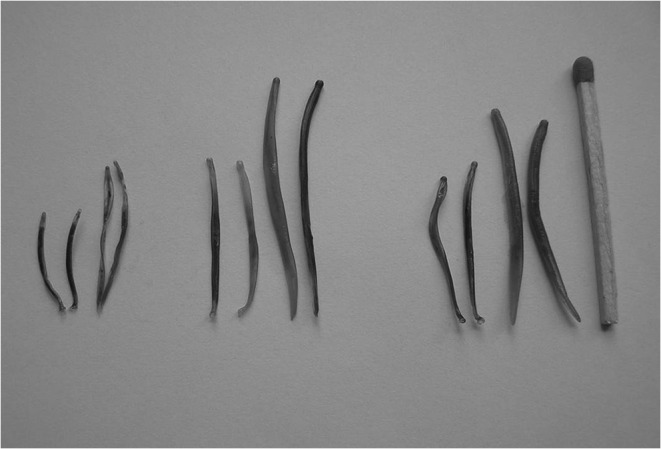
Strongylidae from the genus *Strongylus*: *S. vulgaris*, *S. equinus*, and *S. edentatus*

**Fig. 2:**
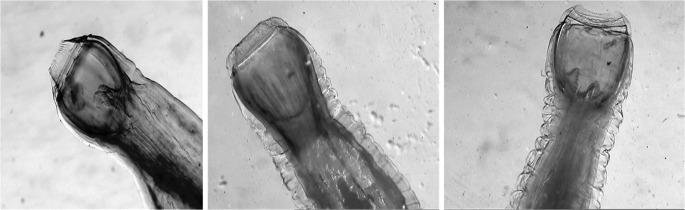
The buccal cavity of large strongyles from the genus *Strongylus*: *S. vulgaris*, *S. edentatus*, and *S. equinus*

The strongyles from the genus *Strongylus* was evidenced in 26.5 % of examined horses (Table 1). Further, it was found that *S. vulgaris* was the dominant strongyle in southeastern Poland with a 22.8 % prevalence whereas the extensiveness of *S. equinus* infestation was markedly lower (1.7 %). *S. edentatus* was found in 18.3 % of examined horses (Figs. 1 and 2). The intensiveness of strongyle infestation from the genus *Strongylus* was low with mean values of 27.2, 12.3, and 7.1 % for *S. vulgaris*, *S. edentatus*, and *S. equinus*, respectively.

**Table 1:** Time-dependent distribution of infestation of strongyles belonging to the genus *Strongylus*

Date	Number of examined horses	Number and percentage (%) of infected horses			
		Strongyles from the genus *Strongylus*	*Strongylus vulgaris*	*Strongylus edentatus*	*Strongylus equinus*
February 2006	35	4 (11.4)	3 (8.6)	2 (5.7)	0
March 2006	40	2 (5.0)	2 (5.0)	2 (5.0)	0
April 2006	85	19 (22.4)	17 (20.0)	11 (12.9)	2 (2.4)
May 2006	97	25 (25.8)	23 (23.7)	17 (17.5)	1 (1.0)
June 2006	56	19 (33.9)	18 (32.1)	12 (21.4)	0
July 2006	78	25 (32.1)	21 (26.9)	16 (20.5)	0
August 2006	Not examined				
September 2006	120	28 (23.3)	21(17.5)	19 (15.8)	0
October 2006	64	23 (35.9)	21 (32.8)	17 (26.6)	0
November 2006	72	20 (27.8)	16 (22.2)	14 (19.4)	4 (5.6)
December 2006	65	19 (29.2)	16 (24.6)	18(27.7)	3 (4.61)
January 2007	55	8 (14.5)	8 (14.5)	5 (9.1)	2 (3.6)
Total	725	192 (26.5)	165 (22.8)	133 (18.4)	12 (1.7)

**Fig. 3:**
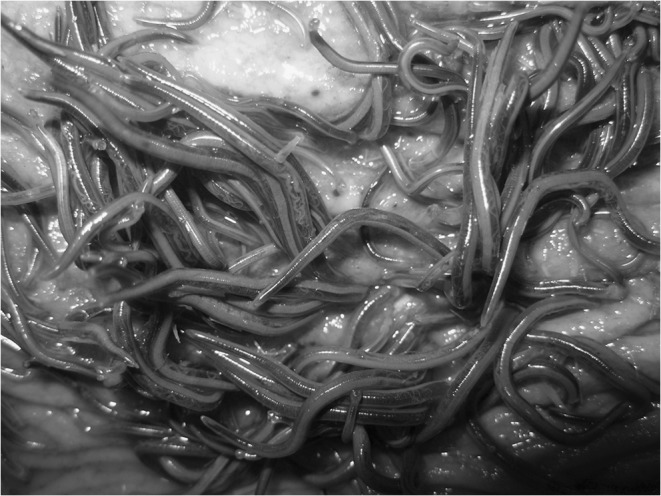
Strongylidae on the mucosal membrane of the cecum

**Fig. 4:**
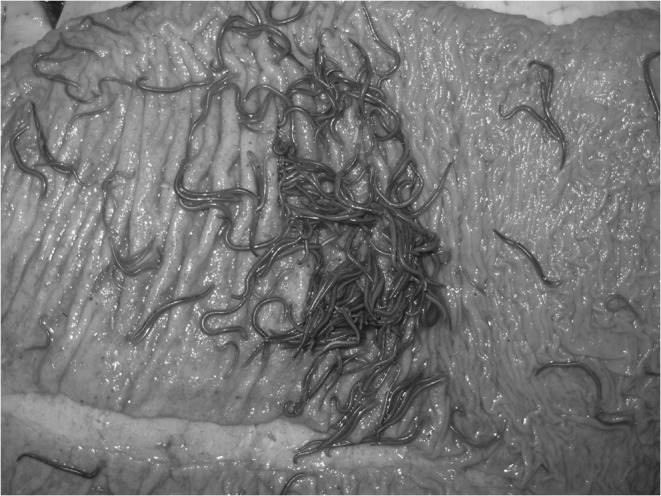
Strongylidae on the mucosal membrane of the abdominal stratum of the large colon

A majority of strongyles belonging to the genus *Strongylus* was attached to the mucosal membrane of the cecum or abdominal stratum of the large colon (Figs. 3 and 4). However, in spite of a relatively low intensiveness, changes in mucosal membranes of several horses were extensive. Numerous hyperemia focuses accompanied by mucosal membrane swellings and extravasations at the site of the parasite location were observed.

The yearly seasonal distribution of the infestation of strongyles belonging to the genus *Strongylus* was shown in Table 1. Seasonal changes in extensiveness of infestation were noted, and the lowest one was found in winter.

Table 2 shows a prevalence of single or multispecies strongyles in examined horses. A dominant combined infestation of *S. vulgaris* and *S. edentatus* was found in 100 of 725 examined horses (52.1 % of animals infected with strongyles).

**Table 2:** Infestation of single- and multispecies strongyles belonging to the genus *Strongylus* in examined horses

Date	Number of horses	Number and percentage (%) of infected horses						
		Single-species			Multispecies			
		*Strongylus vulgaris*	*Strongylus edentatus*	*Strongylus equinus*	*S. vulgaris*	*S. vulgaris*	*S. edentatus*	*S. vulgaris*
					*S. edentatus*	*S. equinus*	*S. equinus*	*S. edentatus*
								*S. equinus*
February 2006	4	2 (50.0)	1 (25.0)	0	1 (25.0)	0	0	0
March 2006	2	0	0	0	2 (100)	0	0	0
April 2006	19	7 (36.8)	1 (5.3)	0	9 (47.3)	1 (5.3)	1 (5.3)	0
May 2006	25	8 (32.0)	2 (8.0)	0	14 (56.0)	0	0	1 (4.0)
June 2006	19	7 (36.8)	1 (5.3)	0	11 (57.9)	0	0	0
July 2006	25	9 (36.0)	4 (16.0)	0	12 (48.0)	0	0	0
August 2006	Not examined							
September 2006	28	9 (32.1)	7 (25.0)	0	12 (42.9)	0	0	0
October 2006	23	6 (26.1)	3 (13.0)	0	14 (60.9)	0	0	0
November 2006	20	5 (25.0)	3 (15.0)	1 (5.0)	8 (40.0)	0	0	3 (15.0)
December 2006	19	1 (5.3)	3 (15.8)	0	12 (63.2)	0	0	3 (15.8)
January 2007	8	1 (12.5)	0	0	5 (62.5)	2 (25.0)	0	0
Total	192	55 (28.7)	25 (13.0)	1 (0.5)	100 (52.1)	3 (1.6)	1 (0.5)	7 (3.7)

## Discussion

There are not many publications considering the genus *Strongylus* because intravital determination of species belonging of the nematode parasites by coproscopy examination is difficult and in some cases impossible. This determination needs comprehensive knowledge and careful morphometric analysis of larvae from fecal cultures or the use of modern molecular methods (Gundłach and Sadzikowski 2004; Nielsen et al. 2008; Schnieder 2006; Traversa et al. 2007).

Not many postmortem examinations of horses focused on prevalence of individual species of strongyles belonging to the genus *Strongylus* performed in Poland indicate *S. vulgaris* as the dominant species. In the present studies involving a large number of animals, the extensiveness of this nematode infestation was 22.8 % making it markedly lower in comparison to a 60 to 78 % prevalence reported by others ((Gawor 1995; Gawor et al. 2006; Gundłach et al. 2004; Sobieszewski 1967). The extensiveness of *S. edentatus* infestation amounted to 18.3 % and was similar to that (20 to 22 %) evidenced earlier in the Lublin Region (Gundłach et al. 2004; Sobieszewski 1967). However, Gawor 1995 found twice higher prevalence amounting to 40 % in the 1990s, but more current studies failed to evidence the nematodes; this controversy may result from the procedure used by the authors (Gawor et al. 2006). Current studies indicating a low prevalence of *S. equinus* (extensiveness, 1.7 %) confirm our earlier observations which failed to find the nematodes in horses from the Lublin Region (Gundłach et al. 2004) and are in contrast to other reports showing *S. equines* in 14 to 28 % of horses (Gawor 1995; Gawor et al. 2006; Sobieszewski 1967).

Comparison of the worldwide distribution of individual strongyles species belonging to strongyles shows *S. vulgaris* as the dominant species with a prevalence amounting up to 88 % (Atlas et al. 2007; Boxell et al. 2003; English 1979; Königová et al. 2001; Kuzmina et al. 2005; Lyons et al. 2006; Pereira and Vianna 2006; Uslu and Guclu 2007). Other species dominated only in few countries, for example *S. edentatus* in Island (Eydal and Gunnarsson 1994) and *S. equinus* in Greece (Theodoridis et al. 1999). However, it should be stressed that the results were obtained using various intravital and postmortem procedures. The examinations included not the same numbers of horses, kept in various systems, used in different ways, and were controlled or not controlled. Moreover, the years in which the examinations were carried out may affect significantly the results, because the strategy of parasite control in horses has been changing since the 1990s.

The extensiveness of infestation of individual strongyles species belonging to the genus *Strongylus* found in the present studies seems to support an opinion suggesting a lowering prevalence in numerous regions of the world. This view is reliably confirmed by the data reported in Germany where *S. vulgaris* and *S. edentatus* were frequently found until recently whereas, now, these nematode parasites occur rarely or are not found in many regions (Schnieder 2006). Similar results were evidenced in Australia indicating a dramatic decrease in prevalence of the genus *Strongylus* during the last decades (Boxell et al. 2003; Bucknell et al. 1995; English 1979). The widespread infestation of strongyles is affected by several variables including the occurrence of drug-resistant strains (Gawor 2006). The lack of drug-resistant strains is a result of long life cycles of these nematode parasites and limited contact of mature nematodes with anthelmintics. The regular use of antiparasitic compounds in horses eliminates these nematodes from infested areas.

The present results indicate that the lowest prevalence of strongyle species, except for *S. equinus*, was found in January, February, and March. However, it is difficult to draw a conclusion because of an extremely low extensiveness of infestation. Similar relationships were reported by others (Chapman et al. 2003). Changes in yearly infestation extensiveness result mainly from the life cycles of strongyles for which the prepatent period may last even a few months (Gundłach and Sadzikowski 2004; Schnieder 2006). Thus, the horses infected on pastures excreted the eggs of strongyles till the next year.

The present results considering the structure of infestation in examined horses with single- or multispecies nematodes belonging to *Strongylus* species seem interesting. These findings indicated that a combined infestation with *S. vulgaris* and *S. edentatus* found in 52.1 % of horses infected with large strongyles was the most frequent. Infestations with one species including *S. vulgaris* (55 horses, 28.7 %) and *S. edentatus* (25 horses, 13 %) were observed less frequently. This tendency persisted for a majority of months in the year.

Results of others (Boxell et al. 2003; Bucknell et al. 1995; English 1979; Schnieder 2006) and our data confirm the view that strongyles belonging to the genus *Strongylus* will occur less frequently in horses. However, these nematode parasites should arouse the veterinary surgeons' interest. Horses severely infected with the nematode parasites exhibit lack of appetite, disorders in the gastrointestinal functioning, and developing emaciation. The large strongyles are a significant reason for colic caused mainly by migrating larvae, especially *S. vulgaris* and also mature parasites. It should be stressed that in the case of nonintensive infestations, sometimes without signs, these parasites decrease the performance and efficiency of horses.
